# Protocol for the development and testing of the schiZotypy Autism Questionnaire (ZAQ) in adults: a new screening tool to discriminate autism spectrum disorder from schizotypal disorder

**DOI:** 10.1186/s12888-023-04690-3

**Published:** 2023-03-28

**Authors:** Rizwan Parvaiz, Erik Vindbjerg, Bernard Crespi, Francesca Happe, Rik Schalbroeck, Zainab Al-Sayegh, Ida-Marie Danielsen, Bruce Tonge, Poul Videbech, Ahmad Abu-Akel

**Affiliations:** 1 Department of ADHD and Autism, Mental Health Services, Capital Region of Denmark, Copenhagen, Denmark; 2 Competence Centre for Transcultural Psychiatry, Mental Health Centre Ballerup, Copenhagen, Denmark; 3 Department of Biological Sciences, Simon Fraser University, Burnaby, BC V5A 1S6 Canada; 4 Social, Genetic & Developmental Psychiatry Centre, Institute of Psychiatry, Psychology & Neuroscience, King’s College London, London, UK; 5 Department of Psychiatry and Neuropsychology, Maastricht University, Maastricht, Netherlands; 6 Centre for Developmental Psychiatry and Psychology, Monash University, Melbourne, Australia; 7 Center for Neuropsykiatrisk Depressionsforskning Psykiatrisk Center Glostrup, Nordstjernevej 41, Glostrup, Copenhagen, 2600 Denmark; 8 School of Psychological Sciences, University of Haifa, 3498838 Haifa, Israel

**Keywords:** Autism Spectrum Disorder (we prefer Autism Spectrum Condition), Schizotypal Disorder, Questionnaire, Diagnostics

## Abstract

**Background:**

Autism spectrum disorder (ASD) and schizotypal disorder (SD) both have a heterogenous presentation, with significant overlaps in symptoms and behaviour. Due to elevated recognition and knowledge of ASD worldwide, there is a growing rate of referrals from primary health professionals to specialised units. At all levels of assessment, the differential diagnostic considerations between ASD and SD exert major challenges for clinicians. Although several validated screening questionnaires exist for ASD and SD, none have differential diagnostic properties. Accordingly, in this study, we aim to develop a new screening questionnaire, the schiZotypy Autism Questionnaire (ZAQ), which provides a combined screening for both conditions, while also indicating the relative likelihood of each.

**Methods:**

We aim to test 200 autistic patients and 100 schizotypy patients recruited from specialised psychiatric clinics and 200 controls from the general population (Phase 1). The results from ZAQ will be compared to the clinical diagnoses from interdisciplinary teams at specialised psychiatric clinics. After this initial testing phase, the ZAQ will be validated in an independent sample (Phase 2).

**Conclusions:**

The aim of the study is to investigate the discriminative properties (ASD vs. SD), diagnostic accuracy, and validity of the schiZotypy Autism Questionnaire (ZAQ).

**Funding:**

Funding was provided by Psychiatric Centre Glostrup, Copenhagen Denmark, Sofiefonden (Grant number: FID4107425), Trygfonden (Grant number:153588), Takeda Pharma.

**Trial registration:**

Clinical Trials, NCT05213286, Registered 28 January 2022, clinicaltrials.gov/ct2/show/NCT05213286?cond = RAADS&draw = 2&rank = 1.

**Supplementary Information:**

The online version contains supplementary material available at 10.1186/s12888-023-04690-3.

## Background

**Fig. 1:**
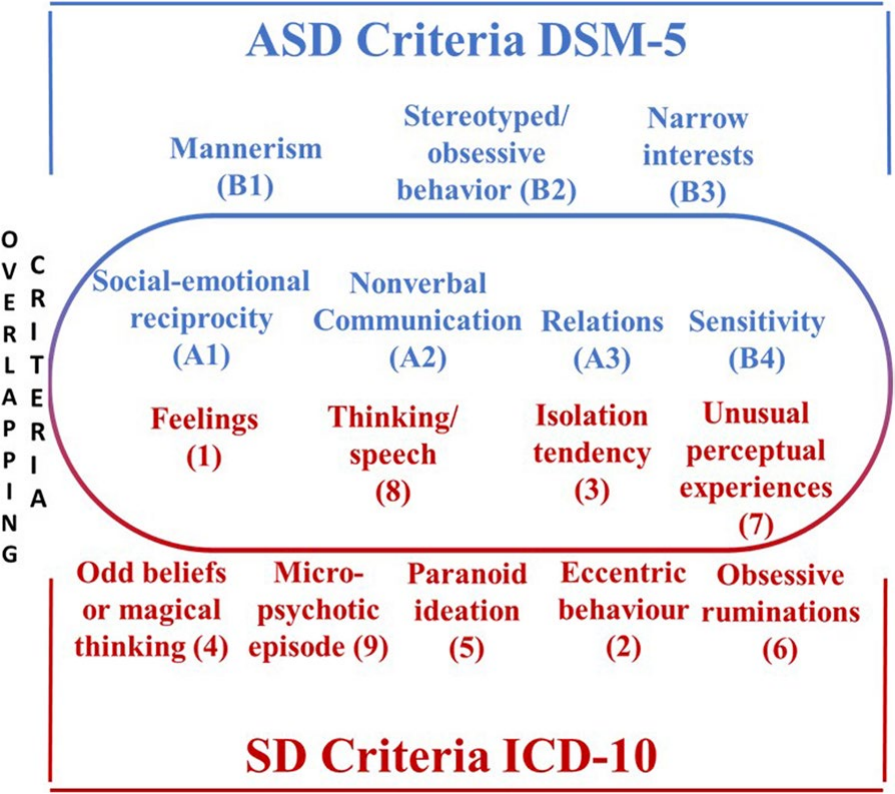
Diagnostic criteria for Autism Spectrum Disorder (ASD) and Schizotypal Disorder (SD). The encircled criteria mark the highest degree of clinical overlap

The psychiatric burden in people with Autism Spectrum Disorder (ASD) [1] is considerably higher compared to neurotypical individuals, with 70% experiencing at least one co-occurring psychiatric disorder and 40% exhibiting two or more psychiatric disorders [2]. In a clinical setting and at all levels of assessment, one of the most difficult differential diagnostic challenges is schizotypal disorder (SD), which shares considerable features with ASD both at the symptomatic level and in the diagnostic criteria (see Fig. 1). Thus, discriminating ASD from SD in a clinical setting is a time-consuming task and demands a high level of expertise of both disorders. An audit of 319 patient cases at our clinic (specialized unit for the assessment of ASD) from January-June 2019 showed that about 37% of the adults referred to our clinic for the assessment of ASD, did not meet diagnostic criteria. Of these, almost 40% were diagnosed on the schizophrenia spectrum (predominantly SD). To improve diagnostic accuracy, it is therefore not only pertinent but also necessary to investigate discriminating features between ASD and SD and to develop tools to aid clinicians in the assessment.

### Autism spectrum disorder

**Fig. 2:**
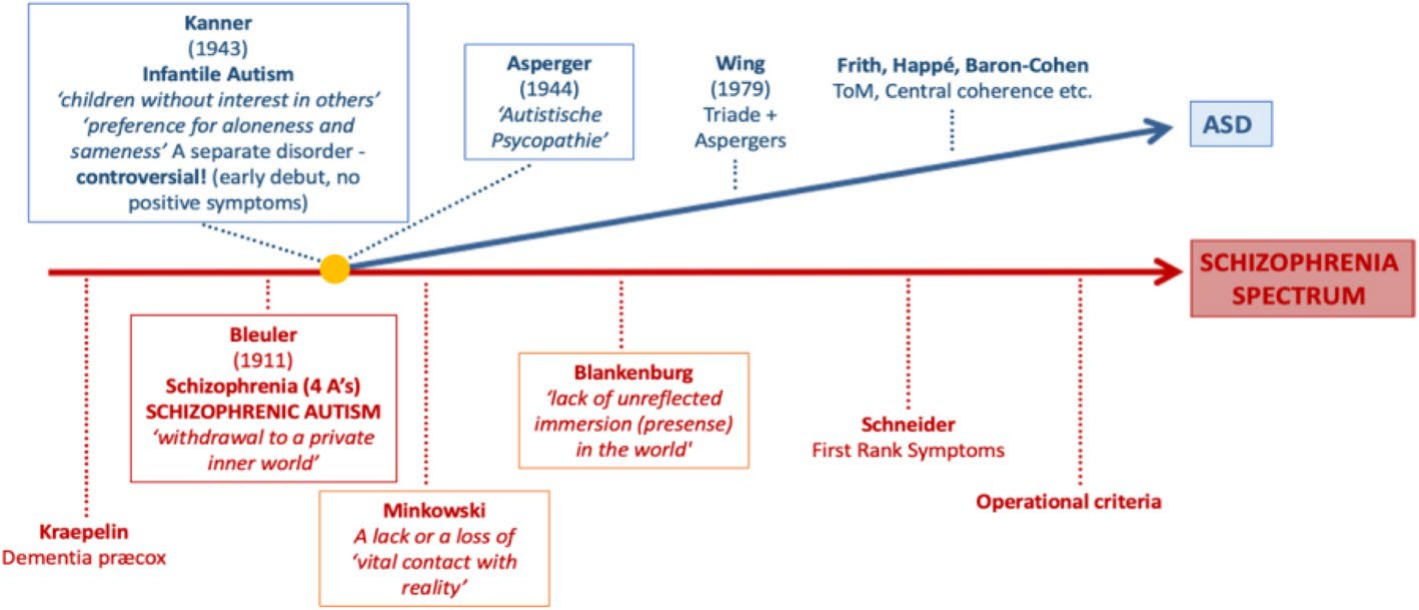
Development of concepts and criteria (figure provided by Maria Nilsson)

ASD and SD historically and practically have been grounded in two different scientific traditions, which has had a significant impact on the way clinicians assess patients and how the diagnostic criteria have developed (see Fig. 2).

ASD has its roots in the relatively young field of child and adolescent psychiatry, whereas SD comes out of a more than 100-year-old tradition and is primarily based on clinical presentations in adults. Consequently, professionals involved in assessment of ASD and SD often come from different backgrounds and make use of different diagnostic methods. The prevalence estimates of ASD display a high variability across nations worldwide. In most western countries the prevalence is estimated to be 1–2% [3]. Danish data suggest a prevalence of 1.65% or approximately 95,000 persons in Denmark [4]. The case identification and assessment of ASD is challenged by a high rate of comorbidity with depression (50%), anxiety (40%) and ADHD/ADD (40–60%) [5, 6]. The prevalence of comorbidity with SD has to our knowledge not been established. Nevertheless, some indications of the relation between ASD and SD can be estimated by looking at studies comparing ASD with psychosis or schizophrenia spectrum disorders (SSD). Sub-clinical psychotic symptoms occur frequently in ASD patients [7–9], and the prevalence of SSD in adults with ASD are in the range of 4–12% [5].

To aid in case identification of ASD, several screening questionnaires have been developed, including the Autism Spectrum Quotient (AQ) [10], the Ritvo Adult Asperger Diagnostic Scale Revised (RAADS-R) [11], and the Social Responsiveness Scale (SRS) [12]. Importantly, however, several studies indicate that such questionnaires do not differentiate well between autistic individuals and individuals with SSD [13, 14].

### Schizotypal disorder

SD is also a heterogeneous disorder with an estimated prevalence of 4.6% and a high rate of comorbidity [15, 16]. The diagnosis can be made if at least 4 out of 9 ICD-10 criteria are met (see Fig. 1). Although debated [17], SD is considered a crucial construct in the development of SSD [18]. Thus, early diagnosis is important for prognosis, as 25–48% of SD patients are prodromal and go on to develop SSD [17, 19]. The prevalence of comorbidity between ASD and SD, which is the main focus of this study, has to our knowledge not been described in any detail. However, the prevalence of ASD in individuals with SSD seems to be substantially increased relative to the general population, although estimates vary widely [20].

Several psychometrically robust questionnaires are available to assess features of and screen for SD. In adult populations these notably include the Schizotypal Personality Questionnaire Brief Revised (SPQ-BR) [21], the Oxford-Liverpool Inventory of Feelings and Experiences (O-LIFE) [22], the Multidimensional Schizotypy Scale (MSS) [23], the Cardiff Anomalous Perception Scale (CAPS) [24], and the Community Assessment of Psychic Experience (CAPE) [25]. In children, these notably include the Melbourne Assessment of Schizotypy in Kids (MASK) [26] and the Schizotypy Personality Questionnaire in Children (SPQ-C) [27], (see Table 2). To our knowledge, none of these have significant psychometric power to discriminate SD from ASD.

Given significant difficulties in discriminating between ASD and SD and the serious consequences that case misclassification can have for treatment and prognosis, we have sought to develop the schiZotypy Autism Questionnaire (ZAQ) to aid clinicians in the assessment of and discrimination between ASD and SD in adults at the case identification stage. A pilot version of this novel screening questionnaire ZAQ, containing 130 questions, has been developed at the Mental Health Centre, Capital Region of Copenhagen. This will be tested in 300 psychiatric patients and 200 healthy controls from May 2022 – April 2023, which constitutes Phase 1 of this study. Psychometric data analysis from this study will constitute the empirical foundation for the development of the final version of ZAQ. This final version, which pending statistical recommendations is expected to contain approximately 60 questions, will undergo subsequent validation in an independent sample (Phase 2).

## Methods

### Description of the creation of ZAQ

To construct the ZAQ, we first performed an exhaustive literature search to identify potential discriminating features between ASD and SD. Complementing the literature search, we interviewed three experienced clinicians who identified discriminating factors based on their clinical knowledge. Altogether, we identified 38 features which appeared to differentiate ASD and SD. These features constituted 11 subscales, subsumed under 6 subdomains (see Table 1). Identification of potentially discriminating features has been the primary focus. Once identified these features were grouped together into well-defined subscales. To present an overview, these subscales are grouped together in subdomains, highlighting the areas showing potential for discrimination [28, 29].

**Table 1:** Summary of the domains, subdomain and subscales serving as the theoretical framework for ZAQ^a^

**Main ZAQ-domains**	**Subdomain**	**Subset of items on which responses between individuals with ASD or SD might differ**	**Subscales**	**Number of questions**
**Diagnostic criteria**	Autism	Stereotyped behavior, narrow interests, hyper-hyposensitivity to sensory stimuli	1. Steretyped behavior	14 questions
			2. Hypersensitivity + Narrow interest	11 questions
	Schizotypy	Self-disorders, hyper-reflexitivity, magical ideation, positive schizotypy	3. Self-disorders	17 questions
			4. Magical ideation + positive schizotypy	13 questions
			5. Disorganised schizotypy	5 questions
			6. Negative schizotypy	10 questions
**Clinical features**	Clinical trajectories	Time of onset of symptoms, childhood symptoms	7. Clinical trajectories and symptoms	19 questions
**Psychological theory**	Theory of Mind (ToM)	Inference of intentions of others, ToM performance, self-referential hypermentalisation, facial emotion perception	8. Theory of Mind	13 questions
	Local vs. global processing	Local vs. global processing, imagination and creativity, reading abilities, word interpretation, savant feature, apophenia	9. Local vs. global processing	8 questions
**Cognitive features**	Attention, memory	Selectivity of attention, selective memory/false memory, working memory performance	10. Cognition	12 questions
			11. Higher Cognitive Functions	9 questions

^a^Note: For a comprehensive account, see also references [28, 29]

Secondly, the research group identified several psychometrically-validated assessment and screening questionnaires for schizotypy and ASD (see Table 2). From this list of questionnaires, questions were extracted based on their clinical face validity, factor loadings from the validity studies of these questionnaires, and reliability scores (e.g., test–retest reliability). This process yielded approximately 950 questions (version 1 ZAQ).

**Table 2:** Scales and questionnaires used in the development of the schiZotypy Autism Questionnaire (ZAQ)

Scales and Questionnaires							
**Scales/ Questionnaires**	**References for validation**	**Scales/ questionnaires**	**References for validation**	**Scales/ questionnaires**	**References for validation**	**Scales/ questionnaires**	**References for validation**
**Schizotypy**		**Theory of Mind**		**Autism Spectrum Disorder (ASD)**		**Cognitive features**	
**SPQ** Schizotypal Personality Questionnaire	Raine 1991 [30]	**TH.O.M.A.S** Theory of Mind Assessment Scale	Bosco et al. 2009 [31]	**RAADS-R** Ritvo Autism Asperger Diagnostic Scale-Revised	Ritvo et al. 2011 [11]	**WMS** Working Memory Scale	Vallat-Azouvi 2012 [32]
**O-LIFE** Oxford-Liverpool Inventory of Feelings and Experiences	Mason et al. 2005 [22]	**Self-referential Hinting Task**	Wastler & Lenzenweger 2019 [33]	**SRS** Social Responsiveness Scale	Constantino et al. 2003 [12]	**DFlex** Detail and Flexibility	Roberts et al. 2011 [34]
**MSS** Multidimensional Schizotypy Scale	Kwapil et al. 2018 [23]	**Strange Stories**	Happe 1994 [35]	**AQ** Autism Spectrum Quotient	Baron-Cohen et al. 2001 [10]	**DOSPERT** Domain Specific Risk Taking	Blais and Weber 2006 [36]
**CAPE** Community Assessment of Psychic Experience	Konings et al. 2006 [25]	**TAS-20** Toronto Alexithymia Scale	Bagby et al. 1994 [37]	**EQ** Empathy Quotient	Baron-Cohen & Wheelwright 2004 [38]	**CRT** Cognitive reflection task	Frederick 2005 [39]
**PAGE-R**	Fach et al. 2013 [40]	**ADI-r** Autism Diagnostic Interview-Revised	Lord et al. 1994 [41]	**RBS** Repetitive Behaviour Scale	Lam el al Aman 2007 [42]	**CAQ** Creative assessment questionnaire	Carson et al. 2005 [43]
**MASK** Melbourne Assessment of Schizotypy in Kids	Jones et al. 2015 [26]	**Self-disorders**		**SPQ** Sensory perception quotient	Tavassoli et al. 2014 [44]	**ARHQ** Adult Reading History Questionnaire	Lefly and Pennington 2000 [45]
**CAPS** Cardiff Anomalous Perception Scale	Bell et al. 2006 [24]	**EASE (Semi Structured)** Examination of Anomalous Self-Experience	Parnas et al. 2005 [46]				
		**SQuEASE6** Short Questionnaire 6 of EASE	Møller 2018 [47]				
		**IPASE** Inventory of Psychotic-Like Anomalous Self-Experiences	Cicero et al. 2019 [48]				

In a first round of evaluation of their face-validity, these 950 questions were presented to six experienced clinicians—each with extensive (+ 5 years) clinical knowledge in either ASD or SD and who were not associated with the research group. These clinicians assessed how their patients would answer these 950 questions. Importantly, questionnaires measuring schizotypy were assessed by clinicians with ASD experience and vice versa. This evaluation retained 195 questions (version 2 ZAQ). All items of the version 2 ZAQ then underwent a second round of evaluation by 5 autistic patients and 2 schizotypy patients. Of these 195 questions, judged on the basis of their unambiguity and clarity, the final 130 questions entering Phase 1 of the study were selected. The resulting subscales ranged from 5–19 items.

### Recruitment and testing

We have designed a multicenter, prospective, non-randomized experimental study, where adult patients will be recruited from May 2022 – April 2023. A group of 300 patients will be recruited from 2 outpatient clinics located at Mental Health Centre Copenhagen: 200 patients (see power calculation) will be recruited from the pool referred to the specialised clinic for diagnostic assessment of ASD at Mental Health Centre Copenhagen, and 100 patients will be recruited from the specialised psychosis clinic at Mental Health Centre with an SD diagnosis. Additionally, a group of 200 adults from the general population without a psychiatric diagnosis will be recruited as a control group. Inclusion and exclusion criteria are detailed in Table 3. The data will be collected on-line and managed through a secure server with full compliance with Personal Data Handling laws of Denmark.

**Table 3:** Inclusion and exclusion criteria

**Inclusion Criteria: Patients 18–65 years (ASD and non-ASD psychiatric)**	**Exclusion Criteria: Patients (ASD and non-ASD psychiatric)**
WAIS-IV or Raven IQ > 85, no-clinical suspicion of intellectual disability and minimum 11 years of education	Insufficient Danish language skills
Completion of: - Clinical Assessment - Adult Asperger Assessment (AAA) - Psychopathology evaluation using DSM-5 - A conclusion based on multidisciplinary consensus	Acute psychiatric illness prompting departure/dismissal from the diagnostic assessment
Controls: Are matched with age, gender, marital status, number of children, and educational level	Controls: Concurrent or prior psychiatric diagnosis (self-report)

**Fig. 3:**
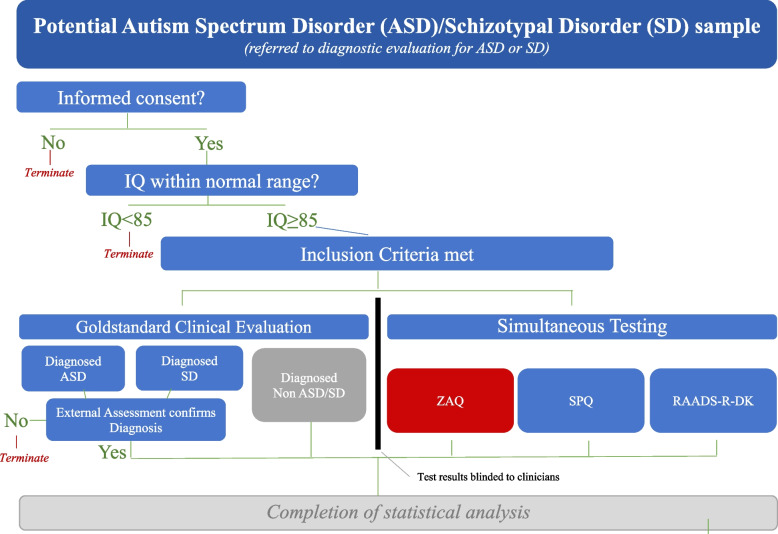
Flow diagram Phase 1 of the ZAQ-study. Patients will undergo clinical evaluation and be divided into 3 groups: Patients with an ASD (Autism Spectrum Disorder) diagnosis, SD (schizotypal Disorder) diagnosis, neither ASD nor SD diagnosis. In parallel and independent from the clinical evaluation, participants will be assessed with the ZAQ schiZotypy Autism Questionnaire), RAADS-R-DK (Ritvo Adult Aspergers diagnostic Schedule – Revised – Danish translation) and SPQ (Schizotypy Personality Questionnaire)

In all three outpatient clinics, the diagnostic assessment will be carried out as usual and will follow gold standard guidelines in their respective fields. The diagnostic assessment of ASD will be performed by clinicians who are not involved in the study. Using DSM 5, the assessment will be based on clinical interviews (minimum 4 h, including childhood history and differential diagnostic assessment) and supplemented by a semi-structured interview (Adult Asperger Assessment, AAA). Video recordings from AAA assessment and the clinical assessment will be presented at a multidisciplinary meeting, with at least 2 experienced clinicians, who will finalize the diagnostic conclusion. A schematic showing the flow of patients is depicted in Fig. 3. The result from this initial phase of the study will undergo psychometric analysis and items displaying adequate validity and discriminating power will be selected for the final ZAQ questionnaire (Phase 2).

### Considerations related to the statistical analysis

#### Statistical models and analyses

##### Factorial evaluation

First, the dimensionality of the ZAQ will be evaluated with confirmatory factor analysis (CFA). If modification indices suggest a better fit from altered subscales, e.g., by relocating individual items to other subscales, these hypotheses will be included in the subsequent unidimensional tests.

##### Unidimensional construct evaluation

Responses to the proposed subscales of the ZAQ will each be analysed with a unidimensional item response theory (IRT) model. The 1-parameter logistic Rasch model represents the gold standard of construct validation and offers stronger inferences than less restricted models. If applicable, violations of the assumption of local dependence and no differential item functioning (DIF) may be accommodated by the extended graphical log-linear Rasch model [49]. If the assumption of *tau* equivalence is violated and this cannot be solved — e.g., by excluding a minimal number of under-discrimination items — a more flexible 2-parameter logistic model may be adapted.

##### Deriving a general factor

The results of the unidimensional results will be used to select an appropriate multidimensional model in which all subscales reflect a general ZAQ trait. If no indications of DIF were found, a higher-order CFA model will be used, specified in line with the validated unidimensional model (e.g., item interaction terms and/or loading restrictions if applicable). Otherwise, a multidimensional IRT model will be used, ensuring an unbiased trait estimation across subgroups.

##### Concurrent validity and screening accuracy

The latent ZAQ estimate will be evaluated against the diagnostic outcome using logistic regression and a receiver operating characteristic (ROC) curve. The predictive power is evaluated by the area under the curve (AUC) and the sensitivity and specificity at their equilibrium, reflecting equal importance of correct identification in both groups. To promote the generalisability of the results, K-fold cross-validation will be used to dynamically divide the sample into training and testing subsamples.

##### Post-hoc analysis

A post-hoc analysis driven by the diagnostic data can increase model performance, while also posing a risk of overfitting the model to the data and thereby reducing the generalizability of the results. To counteract the latter, only modifications considered clinically meaningful by the clinical experts of the research group will be incorporated, and results will either serve purely as hypotheses or will be stabilised with bootstrap aggregation.

In Phase 1, the diagnostic discrimination of each item is evaluated with logistic regression on the diagnostic outcome. Where clinically meaningful, the model will be modified to promote the influence of highly predictive items while also retaining parsimony. The resulting model will serve as a hypothesis in Phase 2.

For the Phase 2 post-hoc analysis, the suitable procedure will depend on the consistency of the ZAQ across Phase 1 and 2. If the ZAQ was mainly shortened, with limited modifications to the items carried over to Phase 2, it will allow for a supervised machine learning based-model on a combined Phase 1 and Phase 2 sample. This analysis will use a random forest algorithm and overfitting will be controlled by bootstrap aggregation and by splitting the sample into training and validation sets. Otherwise, the post-hoc analysis procedure of Phase 1 will be repeated, to inform hypotheses for future studies.

#### Power calculation

Acceptable diagnostic prediction by the ZAQ would manifest as an AUC ≥ 0.7 in the ROC analysis [50]. Establishing this with confidence relies on the variance of the ZAQ trait estimate, which can only be estimated at Phase 1 completion. For the factor analysis, no formal consensus exists for the minimum sample size [51]. While a minimum of *n* = 200 is a popular rule of thumb, Kline [52] suggests an ideal sample-to-item ratio of 20:1 in CFA. This corresponds to *n* ≤ 360 for the separate subscales (≤ 18 items) and 2600 at the aggregate level (130 items). Assuming the ZAQ is abbreviated to 60 items for Phase 2 and taking into account the stabilising effect of pilot testing, we may reasonably consider a combined clinical and non-clinical sample of 500 to provide a ratio of 8:1. This ensures reasonable factorial stability at the aggregate level while offering excellent statistical power at the subscale level.

### Ethical considerations

The study will be conducted in accordance with the Helsinki Declaration 1964, including subsequent revisions. Participants will only be included after signing an informed consent based on oral and written information. The participants may, at any time, choose to withdraw from the study without being required to explain and without affecting the person's future treatment. The study is approved by the Ethical Committee of the Capital Region of Denmark (approval number H-21039423), and the project is reported to the Danish Data Protection Agency. The study investigators are under the impression that the questionnaires and investigations will not lead to any discomfort for the subjects. There are no known expected short- or long-term risks associated with the present study.

### Outcome

We hypothesize that the ZAQ will provide acceptable discrimination between ASD and SD, as indicated by an AUC ≥ 0.7. The ZAQ will retain a clinically meaningful factor structure, instructing further research on distinct sub-constructs. Lastly, the positive predictive value of the cut-off score will have strong clinical power regarding the selection of which patients need further diagnostic examination in either a specialised clinic for the assessment of psychotic disorders or a clinic for the assessment of ASD.

## Discussion

The main incentive to conduct this study has arisen from a clinical need to improve case identification of patients before referring to either ASD or SD assessment. Wrong initial case identification can lead to unacceptable clinical trajectories for the patients. An overlooked prodromal SSD in an autism clinic can delay onset of necessary treatment. A misdiagnosis of a patient in a unit for assessment of SSD can lead to treatment with antipsychotics when not warranted. Thus, incorrect referral can have severe negative consequences for the patient and is cost inefficient. ZAQ is designed to help avoid these unsatisfying trajectories and is primarily developed to alleviate difficulties in the initial case identifying phase of clinical assessment.

## Supplementary Information

**Additional file 1:** **Supplementary file 1.** ZAQ English Version. schiZotypy Autism Questionnaire (ZAQ) in English version.

## Data Availability

The datasets used and/or analyzed during the current study are available from the corresponding author on reasonable request.

## Abbreviations

ZAQ: SchiZotypi Autism Questionnaire
ASD: Autism Spectrum Disorder
SD: Schizotypal Disorder
ADHD: Attention Deficit Hyperactivity Disorder
ADD: Attention Deficit Disorder
SSD: Schizophrenia Spectrum Disorder
RAADS-R: Ritvo Adult Asperger Diagnostic Scale Revised
SRS: Social Responsiveness Scale
SPQ-BR: Schizotypal Personality Questionnaire Brief Revised
O-LIFE: Oxford-Liverpool Inventory of Feelings and Experiences
MMS: Multidimensional Schizotypy Scale
CAPS: Cardiff Anomalous Perception Scale
CAPE: Community Assessment of Psychic Experience
MASK: Melbourne Assessment of Schizotypy in Kids (MASK)
SPQ-C: Schizotypy Personality Questionnaire in Children
Tom: Theory of Mind
CFA: Confirmatory Factor Analysis
IRT: Item Response Theory
DIF: Differential Item Functioning
RUC: Receiver Operating Characteristics
AUC: Area under the Curve
