# Supplementary material for: Protocol for the development and testing of the schiZotypy Autism Questionnaire (ZAQ) in adults: a new screening tool to discriminate autism spectrum disorder from schizotypal disorder
Source: BMC Psychiatry. 2023 Mar 28;23:200. doi: 10.1186/s12888-023-04690-3 (PMC10044373; doi:10.1186/s12888-023-04690-3)
Supplement: Supplementary file 1 — Additional file 1: Supplementary file 1. ZAQ English Version. schiZotypy Autism Questionnaire (ZAQ) in English version. [file 12888_2023_4690_MOESM1_ESM.docx]

**ZAQ English Version**

| Questions original english version | Response categories |
| --- | --- |
| 1. I prefer not to break the law, no matter how minor. | 1, Strongly disagree 2, Disagree  3, Agree  4, Strongly agree |
| 1. I dislike when other people touch me when we are talking (for example if they lay a hand on my shoulder). | 1 = strongly disagree 2 = disagree  3 = agree  4 = strongly agree |
| 1. I sometimes have the feeling that I am watching myself from outside my body. | 1 = strongly disagree 2 = disagree  3 = agree  4 = strongly agree |
| 1. I have sometimes felt that other people were able to read my mind. | 1 = strongly disagree 2 = disagree  3 = agree  4 = strongly agree |
| 1. It is difficult for me to understand social “rules” e.g., at family gatherings or with a group of friends. Therefore, I either don’t go at all or I get very anxious before going. | 1 = strongly disagree 2 = disagree  3 = agree  4 = strongly agree |
| 1. Negotiating is difficult for me in many situations e.g., prices at flea markets, buying/selling used cars, clothes or electronics, or negotiating salary in a job interview. | 1 = strongly disagree 2 = disagree  3 = agree  4 = strongly agree |
| 1. I can easily tell if someone else is interested or bored with what I am saying and I easily adjust to this. | 1 = strongly disagree 2 = disagree  3 = agree  4 = strongly agree |
| 1. I usually see patterns in many things that other people don't notice. | 1 = strongly disagree 2 = disagree  3 = agree  4 = strongly agree |
| 1. I find it easy to point out a friend in the crowd. | 1 = strongly disagree 2 = disagree  3 = agree  4 = strongly agree |
| 1. I always want to be seated in the same spot. | 1 = strongly disagree 2 = disagree  3 = agree  4 = strongly agree |
| 1. When I am at home during the day, I keep the shades or curtains down, because I am bothered by the light. | 1 = strongly disagree 2 = disagree  3 = agree  4 = strongly agree |
| 1. Sometimes I feel that I am not really present in this world. | 1 = strongly disagree 2 = disagree  3 = agree  4 = strongly agree |
| 1. I believe that ghosts or spirits can influence my life. | 1 = strongly disagree 2 = disagree  3 = agree  4 = strongly agree |
| 1. I often feel so disconnected from the world that it is difficult for me to do things. | 1 = strongly disagree 2 = disagree  3 = agree  4 = strongly agree |
| 1. If I’m invited to a party, without knowing the specifics of it (e.g., are the plans to eat, dance or just relax? How many people will be coming? Who? What is the menu and seating arrangement?), I get anxious, and it is difficult for me to participate. | 1 = strongly disagree 2 = disagree  3 = agree  4 = strongly agree |
| 1. When I am home alone without any plans, most of the time I am relaxed and mentally undisturbed. | 1 = strongly disagree 2 = disagree  3 = agree  4 = strongly agree |
| 1. I can sense if I am being intrusive even if the other person doesn’t tell me. | 1 = strongly disagree 2 = disagree  3 = agree  4 = strongly agree |
| 1. I think I sometimes bore others, when I go into details about the things I talk about. | 1 = strongly disagree 2 = disagree  3 = agree  4 = strongly agree |
| 1. It is very important, almost necessary, for me to make a shopping list before I go grocery shopping. | 1 = strongly disagree 2 = disagree  3 = agree  4 = strongly agree |
| 1. In primary school, I had better math skills than most of my fellow students. | 1 = strongly disagree 2 = disagree  3 = agree  4 = strongly agree |
| 1. When I am at social occasions, I find myself repeating the same topics or questions,  especially if I previously have received a positive response doing so. | 1 = strongly disagree 2 = disagree  3 = agree  4 = strongly agree |
| 1. I am sensitive to smells. E.g. I leave or move to another section when I smell a strong odour in a store (for example, bath products, candles, perfumes). | 1 = strongly disagree 2 = disagree  3 = agree  4 = strongly agree |
| 1. Sometimes when I look in the mirror my face seems quite different from usual. | 1 = strongly disagree 2 = disagree  3 = agree  4 = strongly agree |
| 1. I am worried that people on other planets may be influencing what happens on Earth. | 1 = strongly disagree 2 = disagree  3 = agree  4 = strongly agree |
| 1. Sometimes I observe that my experience of time changes dramatically. That time passes much faster or slower than usual. | 1 = strongly disagree 2 = disagree  3 = agree  4 = strongly agree |
| 1. It is difficult for me to understand what my family or acquaintances mean during conversations, and I feel misunderstood, which is why it is hard for me to be with them. | 1 = strongly disagree 2 = disagree  3 = agree  4 = strongly agree |
| 1. I often overthink the social encounters in my life (both the ones I have had earlier in life and the upcoming), to such a degree that it makes my daily routines or my job/education difficult to carry out. | 1 = strongly disagree 2 = disagree  3 = agree  4 = strongly agree |
| 1. As a child I struggled compared to my peers with knowing how to get along with other children. | 1 = strongly disagree 2 = disagree  3 = agree  4 = strongly agree |
| 1. I find it difficult to write concisely: I often exceed the numbers of words allowed and find it difficult to decide which details should be left out. | 1 = strongly disagree 2 = disagree  3 = agree  4 = strongly agree |
| 1. Earlier in my life, I have had strange (illogical) experiences, where the conception of my surroundings and my sense of self changed significantly, which made me feel insecure and stressed. As a result, I became more withdrawn and avoided being with others. | 1 = strongly disagree 2 = disagree  3 = agree  4 = strongly agree |
| 1. I have more talent or skill than the average person within at least one of the following areas: visual art (painting, drawing), music (singing or playing), scientific experimentation, mathematics or computer science. | 1 = strongly disagree 2 = disagree  3 = agree  4 = strongly agree |
| 1. In my teenage years, I experienced a change in myself, where I often withdrew into my own thoughts, and became occupied by them, for better or worse. I became less interested in being social and spent more time by myself. | 1 = strongly disagree 2 = disagree  3 = agree  4 = strongly agree |
| 1. I have a tendency to think a lot about philosophical questions (like the meaning of life, or life after death) or metaphysical questions (like, are we able to understand reality, are we alone in the universe). | 1 = strongly disagree 2 = disagree  3 = agree  4 = strongly agree |
|  |  |
| 1. If given a choice about what to eat, how and which route of transport to choose or what song or TV-show/film to watch, I would feel a strong urge to choose what I am used to, rather than choosing something new. | 1 = strongly disagree 2 = disagree  3 = agree  4 = strongly agree |
| 1. Sometimes I experience unusual burning sensations or other strange feelings in or on my body, which cannot be explained by a physical illness | 1 = strongly disagree 2 = disagree  3 = agree  4 = strongly agree |
| 1. I have sometimes experienced that my thoughts about being alive and life are confusing. Where I often think a lot about ordinary things like: why the grass is green, why the traffic lights have three colours or why we have two eyes? | 1 = strongly disagree 2 = disagree  3 = agree  4 = strongly agree |
| 1. Some people can make me aware of them just by thinking about me. | 1 = strongly disagree 2 = disagree  3 = agree  4 = strongly agree |
| 1. During my adolescence, I experienced that I gradually preferred to speak less with people around me, becoming more withdrawn, with a tendency to isolate myself from others. | 1 = strongly disagree 2 = disagree  3 = agree  4 = strongly agree |
| 1. As a child, I played with toys in an unusual way (unscrewed the wheels of a toy car but didn’t play with it like a toy car or played with a doll’s hair, but not the whole doll). | 1 = strongly disagree 2 = disagree  3 = agree  4 = strongly agree |
| 1. I am more challenged than most people when trying to understand other people’s perspectives. | 1 = strongly disagree 2 = disagree  3 = agree  4 = strongly agree |
| Choose the number 1 and 7 | 1 = 0  2 = 1  3 = 2  4 = 3  5 = 4  6 = 5  7 = 6  8 = 7  9 = 8 |
| 1. You and your friend have plans to study for an upcoming exam. Your friend arrives at your place after walking across campus in the freezing cold. You are anxious about the exam, so you immediately start reviewing the material, but your friend interrupts you saying, “brrr it’s so cold outside, I can barely feel my fingers”. | 1 = Your friend is more concerned about the cold than helping you study.  2 = Your friend wants you to know it’s cold outside.  3 = Your friend is freezing and wants to get warm before studying.  4 = None of the above. |
| 1. I often feel vulnerable and unsafe as I am unable to see threats (or opportunities) that are out of my field of vision. | 1 = strongly disagree 2 = disagree  3 = agree  4 = strongly agree |
| 1. It is important for me to know exactly what my plans are for the upcoming days so I make detailed schedules, that I am compelled to follow. | 1 = strongly disagree 2 = disagree  3 = agree  4 = strongly agree |
| 1. When I look at ordinary things like trees or clouds, I often search for patterns. | 1 = strongly disagree 2 = disagree  3 = agree  4 = strongly agree |
| 1. I have sometimes experienced that my thoughts or feelings are located at a specific place in my head. | 1 = strongly disagree 2 = disagree  3 = agree  4 = strongly agree |
| 1. In my teenage years, I experienced a change in my mood and emotions, where the positive feelings gradually became significantly toned down (downplayed). | 1 = strongly disagree 2 = disagree  3 = agree  4 = strongly agree |
| 1. As a child/preteen I heard voices  that were not based on reality (not coming from a person I could see, TV, radio etc.). | 1 = strongly disagree 2 = disagree  3 = agree  4 = strongly agree |
| 1. I take things too literally and therefore misunderstand the actual meaning in the conversation. | 1 = strongly disagree 2 = disagree  3 = agree  4 = strongly agree |
| 1. You just got back from a weekend trip visiting family. When you return you leave your suitcase in the common room of your apartment/dorm even though you know your roommate prefers that you keep your personal belongings in your room. Your roommate notices your suitcase and asks if you are going on another trip. | 1 = Your roommate prefers to have the apartment / college to himself.  2 = Your roommate is curious if you should leave again.  3 = Your roommate wants you to put your suitcase in your room.  4 = None of the above. |
| 1. I have the tendency to focus on details and patterns to a degree where I lose the overall point (for example focusing on colors or details in the background og lose the plot). | 1 = strongly disagree 2 = disagree  3 = agree  4 = strongly agree |
| 1. When I see physical objects move around, they almost seem alive. | 1 = strongly disagree 2 = disagree  3 = agree  4 = strongly agree |
| 1. Sometimes I have difficulty telling whether I am experiencing something or just imagining it. | 1 = strongly disagree 2 = disagree  3 = agree  4 = strongly agree |
| 1. I have many times experienced that the sensory signals from the outside world are distorted (vague, unclear, hazy). | 1 = strongly disagree 2 = disagree  3 = agree  4 = strongly agree |
| 1. Sometimes, I have the feeling that there is a person or force around me, even though I can’t see anybody nearby. | 1 = strongly disagree 2 = disagree  3 = agree  4 = strongly agree |
| 1. My thoughts are so hazy and unclear that I wish that I could just reach up and put them into place. | 1 = strongly disagree 2 = disagree  3 = agree  4 = strongly agree |
| 1. During my adolescence, I gradually (from months to years) experienced a change in my mood, where I became more superficial and stopped reacting to the news with the same sadness or joy as before. | 1 = strongly disagree 2 = disagree  3 = agree  4 = strongly agree |
| 1. As a child/preteen I had paranoid or suspicious ideas about other people’s behaviour and motives. | 1 = strongly disagree 2 = disagree  3 = agree  4 = strongly agree |
| 1. When I look at people outside from inside my house, I predominantly perceive them as physical or biological objects and less as human beings with intentions. | 1 = strongly disagree 2 = disagree  3 = agree  4 = strongly agree |
| 1. You and your roommate usually walk to class together. You are getting ready in the bathroom when your roommate frantically runs out of the bedroom saying: “I’m running late. Did you hear my alarm go off?” | 1 = Your roommate blames you for being late.  2 = Your roommate will know if his / her alarm has rung.  3 = Your roommate wonders why you did not wake him / her.  4 = None of the above. |
| 1. I get preoccupied by one or more parts of objects instead of the whole object, for example buttons on clothes, wheels on cars or dimples on cheeks. | 1 = strongly disagree 2 = disagree  3 = agree  4 = strongly agree |
| 1. Sometimes I’m unsure whether my feelings, thoughts, sensations or actions are completely my own. | 1 = strongly disagree 2 = disagree  3 = agree  4 = strongly agree |
| 1. I have experienced that my interests changed significantly compared to earlier, so that I suddenly became very preoccupied with existential, religious, or supernatural themes. | 1 = strongly disagree 2 = disagree  3 = agree  4 = strongly agree |
| 1. I sometimes hear my own thoughts spoken so loud in my head, that someone nearby might be able to hear them. | 1 = strongly disagree 2 = disagree  3 = agree  4 = strongly agree |
| 1. Earlier in my life, I experienced losing control over my thoughts or my emotions, which made me feel different, insecure and confused. As a result, I had fewer social interactions and became more withdrawn. | 1 = strongly disagree 2 = disagree  3 = agree  4 = strongly agree |
| 1. I have sometimes experienced that there is a hidden meaning, or message to me, in everything that happens around me, as if all things or events around me seem to have to do with me. | 1 = strongly disagree 2 = disagree  3 = agree  4 = strongly agree |
|  |  |
| 1. I have in periods throughout my life been occupied with collecting specific items. | 1 = strongly disagree 2 = disagree  3 = agree  4 = strongly agree |
| 1. I’m uncomfortable wearing certain fabrics (for example, wool, silk, corduroy, tags in clothing). | 1 = strongly disagree 2 = disagree  3 = agree  4 = strongly agree |
| 1. Sometimes when I read, I feel like the words are being read by someone else. | 1 = strongly disagree 2 = disagree  3 = agree  4 = strongly agree |
| 1. I worry that other people are out to get me. | 1 = strongly disagree 2 = disagree  3 = agree  4 = strongly agree |
| 1. Most of the time I find it very difficult to get my thoughts in order. | 1 = strongly disagree 2 = disagree  3 = agree  4 = strongly agree |
| 1. Earlier in my life, I experienced a permanent reduction or loss of ability to feel joy, where the world around me and life in general turned grey or felt empty. | 1 = strongly disagree 2 = disagree  3 = agree  4 = strongly agree |
| 1. I am able to mediate diplomatically between two parties or people with a dispute. For example, between husband and wife, colleagues at work or political opponents. | 1 = strongly disagree 2 = disagree  3 = agree  4 = strongly agree |
| 1. I find it more difficult than other people to recognize if someone means something different from what they are saying. | 1 = strongly disagree 2 = disagree  3 = agree  4 = strongly agree |
| 1. I often focus on details when reading rather than understanding the gist. | 1 = strongly disagree 2 = disagree  3 = agree  4 = strongly agree |
| 1. I get very distressed if plans get changed at the last minute. | 1 = strongly disagree 2 = disagree  3 = agree  4 = strongly agree |
| 1. Even people who do not know me well, are able to sense my desires and respond to them. | 1 = strongly disagree 2 = disagree  3 = agree  4 = strongly agree |
| 1. A bat and a ball cost $1.10 in total. The bat costs $1.00 more than the ball. How much does the ball cost? | 1 = 1.00 $ 2 = 0.10 $  3 = 0.05 $  4 = 0.50 $ |
| 1. I don't have a problem with forgiving or forgetting other people's mistakes. | 1 = strongly disagree 2 = disagree  3 = agree  4 = strongly agree |
| 1. When I hear certain sounds, voices, or music, with my eyes closed, I see colours, shapes, or letters, inside of me. | 1 = strongly disagree 2 = disagree  3 = agree  4 = strongly agree |
| 1. Sometimes it feels like my legs, arms, or other parts of my body are not really mine. | 1 = strongly disagree 2 = disagree  3 = agree  4 = strongly agree |
| Choose the number 3 and 5 | 1 = 0  2 = 1  3 = 2  4 = 3  5 = 4  6 = 5  7 = 6  8 = 7  9 = 8 |
| 1. I have sometimes felt a sense of being completely unique and extraordinary in the world - as if I were the centre of the universe. | 1 = strongly disagree 2 = disagree  3 = agree  4 = strongly agree |
| 1. I believe that I could read other peoples' minds if I really tried. | 1 = strongly disagree 2 = disagree  3 = agree  4 = strongly agree |
| 1. Most of the time before making a decision, I thoroughly weigh the pros and cons. | 1 = strongly disagree 2 = disagree  3 = agree  4 = strongly agree |
| 1. If it takes 5 machines 5 minutes to make 5 widgets, how long would it take 100 machines to make 100 widgets? | 1 = 1 minute 2 = 10 minutes  3 = 5 minutes  4 = 100 minutes |
| 1. I have periods in my life where I intensively collect information about specific things (for example car brands, bird species, types of planets, historical persons). I do this especially when I have to buy things. | 1 = strongly disagree 2 = disagree  3 = agree  4 = strongly agree |
| 1. Sometimes when I am thinking, my thoughts seem so loud that I wonder if other people can hear them. | 1 = strongly disagree 2 = disagree  3 = agree  4 = strongly agree |
| 1. I have sometimes felt like the world is unreal, an illusion or a deception. | 1 = strongly disagree 2 = disagree  3 = agree  4 = strongly agree |
| 1. I have sometimes felt as if things written in magazines or things said on TV were directed towards me. | 1 = strongly disagree 2 = disagree  3 = agree  4 = strongly agree |
|  |  |
| 1. I like to have the same daily routine in household chores, school or at work and I get stressed if they change. | 1 = strongly disagree 2 = disagree  3 = agree  4 = strongly agree |
| 1. I don’t like particular food textures (for example, peaches with skin, applesauce, cottage cheese). | 1 = strongly disagree 2 = disagree  3 = agree  4 = strongly agree |
| 1. I believe that the accidents in my life are caused by mysterious forces. | 1 = strongly disagree 2 = disagree  3 = agree  4 = strongly agree |
| 1. When I have difficulties solving a problem, I usually ask people for advice or assistance. | 1 = strongly disagree 2 = disagree  3 = agree  4 = strongly agree |
| 1. I had more difficulty learning to read in primary school compared with other students my age | 1 = strongly disagree 2 = disagree  3 = agree  4 = strongly agree |
|  |  |
| 1. As a child, I often didn’t look people directly in the eyes, except for my parents. | 1 = strongly disagree 2 = disagree  3 = agree  4 = strongly agree |
| 1. As a child, I did not react when others wanted me to focus my attention on the same thing as them. E.g., when someone pointed at an object or a person, I did not focus on what was being pointed at. | 1 = strongly disagree 2 = disagree  3 = agree  4 = strongly agree |
| 1. As a child, when other people smiled at me I didn’t smile back. | 1 = strongly disagree 2 = disagree  3 = agree  4 = strongly agree |
| 1. As a child or in adult life, I have had stereotypical behavior like clapping my hands, spinning around or had especially stiff and repetitive movements when excited or stressed. | 1 = strongly disagree 2 = disagree  3 = agree  4 = strongly agree |
| 1. As a child, I liked playing with the same toy for extended periods (many hours). | 1 = strongly disagree 2 = disagree  3 = agree  4 = strongly agree |
| 1. As a child I often bumped into people when I was walking on the streets or in a crowd. | 1 = strongly disagree 2 = disagree  3 = agree  4 = strongly agree |
| 1. As a child I sometimes used my toys as real people. In other words, I played with my dolls and made them pour coffee and serve it or got my action figures to go out to the car and drive it. | 1 = strongly disagree 2 = disagree  3 = agree  4 = strongly agree |
| 1. As a child I experienced vivid daydreams. | 1 = strongly disagree 2 = disagree  3 = agree  4 = strongly agree |
| 1. As a child, I found it difficult participating in pretend play, like playing mummy and daddy, or playing cowboys and indians. | 1 = strongly disagree 2 = disagree  3 = agree  4 = strongly agree |
| 1. As a child I believed that I had super- or magical powers. | 1 = strongly disagree 2 = disagree  3 = agree  4 = strongly agree |
| 1. As a child I struggled compared to my peers with understanding other kids’ jokes, sarcasm or deception. | 1 = strongly disagree 2 = disagree  3 = agree  4 = strongly agree |
| 1. I have extraordinary skills (savant-like) e.g., in drawing (hyper-detailed drawings), in maths (fast mental arithmetic), in calendar calculation (the ability to provide the day of the week for any given date) and an exceptional ability to recall the details of facts or events. | 1 = strongly disagree 2 = disagree  3 = agree  4 = strongly agree |
| 1. I would never consider riding a motorcycle without a helmet | 1 = strongly disagree 2 = disagree  3 = agree  4 = strongly agree |
| 1. I find it difficult to remember the story line in films, plays, or books, but I can remember specific scenes in great detail. | 1 = strongly disagree 2 = disagree  3 = agree  4 = strongly agree |
| 1. I would without hesitation consider letting a consultant at the travel agency plan my holiday trip. | 1 = strongly disagree 2 = disagree  3 = agree  4 = strongly agree |
| 1. When others around me turn their attention to something, I become interested in it. | 1 = strongly disagree 2 = disagree  3 = agree  4 = strongly agree |
| 1. I believe that there are secret signs in the world if you just know how to look for them. | 1 = strongly disagree 2 = disagree  3 = agree  4 = strongly agree |
| 1. I like being by myself with my hobbies, rather than spending time with other people. | 1 = strongly disagree 2 = disagree  3 = agree  4 = strongly agree |
| 1. I use strategies to drown out sound (for example, close the door, cover my ears or wear ear plugs in public). | 1 = strongly disagree 2 = disagree  3 = agree  4 = strongly agree |
| 1. In discussions it is difficult for other people to persuade me of an alternative point of view. | 1 = strongly disagree 2 = disagree  3 = agree  4 = strongly agree |
| 1. I have sometimes had an experience that there is something magical or supernatural in the surroundings - or that we exist in a parallel universe. | 1 = strongly disagree 2 = disagree  3 = agree  4 = strongly agree |
| 1. In a lake, there is a patch of lily pads. Every day, the patch doubles in size. If it takes 48 days for the patch to cover the entire lake, how long would it take for the patch to cover half of the lake? | 1 = 24 days  2 = 10 days  3 = 12 days  4 = 47 days |
| 1. I have a higher reading speed than others of the same age and education level. | 1 = strongly disagree 2 = disagree  3 = agree  4 = strongly agree |
| 1. I would not hesitate to pass off somebody else’s work as my own. | 1 = strongly disagree 2 = disagree  3 = agree  4 = strongly agree |
| 1. I dislike being in a situation where I need to make quick decisions. | 1 = strongly disagree 2 = disagree  3 = agree  4 = strongly agree |
| 1. Every time I buy new clothes and shoes, I prefer to buy the same brands and colours. | 1 = strongly disagree 2 = disagree  3 = agree  4 = strongly agree |
| 1. Most of the time I correct people, when they do something wrong. | 1 = strongly disagree 2 = disagree  3 = agree  4 = strongly agree |
| Choose the number 0 + 8 | 1 = 0  2 = 1  3 = 2  4 = 3  5 = 4  6 = 5  7 = 6  8 = 7  9 = 8 |
| 1. When I am listening to music with others (friends, family or partner), I do not get bored of listening to the same song(s) over and over again, whereas they prefer to change songs or vary the song list. | 1 = strongly disagree 2 = disagree  3 = agree  4 = strongly agree |
| 1. I often wonder if everyone in the world is part of a secret experiment. | 1 = strongly disagree 2 = disagree  3 = agree  4 = strongly agree |
| 1. One or both of my parents work or have an education in the field of | 1 = Science or maths  2 = Engineering  3 = IT  4 = None of the above |
| 1. I would drive a car without wearing a seatbelt. | 1 = strongly disagree 2 = disagree  3 = agree  4 = strongly agree |
| 1. I have  more difficulty remembering addresses, phone numbers, or dates, compared with other people my age. | 1 = strongly disagree 2 = disagree  3 = agree  4 = strongly agree |
| 1. I like to collect things and organize them by their size, colour, brand etc. | 1 = strongly disagree 2 = disagree  3 = agree  4 = strongly agree |
| 1. I sometimes hear voices when I am alone (not coming from the radio, TV, telephone etc.). | 1 = strongly disagree 2 = disagree  3 = agree  4 = strongly agree |
| 1. I have a hard time coming up with new ideas (for dinner, places to go with friends, vacation activities). | 1 = strongly disagree 2 = disagree  3 = agree  4 = strongly agree |
| 1. How much time do you spend on average on social media like Facebook, Instagram, Twitter, BeReal, Snapchat etc.? | 1 = 5-15 minutes daily  2 = 15-60 minutes daily  3 = More than 1 hour daily  4 = Less than 5 minutes or very seldom |
| 1. How often do you smoke? | 1 = I smoke everyday  2 = I smoke on most days  3 = I smoke on some days  4 = I do not smoke at all |
